# Ecological Consequences of Clonal Integration in Plants

**DOI:** 10.3389/fpls.2016.00770

**Published:** 2016-06-30

**Authors:** Fenghong Liu, Jian Liu, Ming Dong

**Affiliations:** ^1^Institute of Botany, Chinese Academy of SciencesBeijing, China; ^2^National Science Library, Chinese Academy of SciencesBeijing, China; ^3^Institute of Environmental Research, Shandong UniversityJinan, China; ^4^State Key Laboratory of Vegetation and Environmental Change, Institute of Botany, Chinese Academy of SciencesBeijing, China; ^5^Key Laboratory of Hangzhou City for Ecosystem Protection and Restoration, Hangzhou Normal UniversityHangzhou, China

**Keywords:** clonal plants, environmental heterogeneity, physiological integration, ramet/genet, resource translocation

## Abstract

Clonal plants are widespread throughout the plant kingdom and dominate in diverse habitats. Spatiotemporal heterogeneity of environment is pervasive at multiple scales, even at scales relevant to individual plants. Clonal integration refers to resource translocation and information communication among the ramets of clonal plants. Due to clonal integration, clonal plant species possess a series of peculiar attributes: plasticity in response to local and non-local conditions, labor division with organ specialization for acquiring locally abundant resources, foraging behavior by selective placement of ramets in resource-rich microhabitats, and avoidance of intraclonal competition. Clonal integration has very profound ecological consequences for clonal plants. It allows them to efficiently cope with environmental heterogeneity, by alleviating local resource shortages, buffering environmental stresses and disturbances, influencing competitive ability, increasing invasiveness, and altering species composition and invasibility at the community level. In this paper, we present a comprehensive review of research on the ecological consequences of plant clonal integration based on a large body of literature. We also attempt to propose perspectives for future research.

## Introduction

Modularity occurs in almost all vascular plants (Watkinson and White, 1986; Dong, 1996a,b; Clarke, 2012). Plants can be regarded as an assembly of many modules (de Kroon et al., 2005). When their modules are capable of iterating themselves in a spontaneous way, and thus produce potentially independent offspring through vegetative propagation, the plants are referred to as clonal plants (Mogie and Hutchings, 1990; Dong, 1996a,b, 2011; de Kroon and van Groenendael, 1997; Hutchings and Wijesinghe, 1997). In clonal plants, the clonally formed offspring are specifically referred to ‘ramets,’ a term coined by Harper (1977). The whole plant, which is often comprised of a number of ramets of the same clone, is referred to as a ‘genet’ (Harper, 1977). Different ramets belonging to the same genet will have actually developed from a single zygote, and thus share the same genotype (Harper, 1977, 1985; Clarke, 2012). Within a genet, each ramet has the potential to perform all biological functions as an independent, non-clonal plant, even if it is separated from the rest of the genet (Hutchings and Wijesinghe, 1997). In this respect, a grown-up ramet can be regarded as an individual.

It is well-understood that various materials including external resources absorbed by plants, hormones, photosynthates, and secondary metabolites can be translocated among different parts of an intact plant individual via its interconnected vascular structures (Bray, 1954; van Groenendael and de Kroon, 1990). Ramets of clonal plants normally stay connected through horizontal connecters (stolons and rhizomes) for an extended period, enabling physiological integration between ramets (Dong, 2011). Experiments using isotope tracers (^13^C or ^14^C, and D) and acid fuchsine to investigate the patterns of resource translocation within clonal plant species have provided direct evidence to confirm this possibility (Guttridge, 1959; Qureshi and Spanner, 1971; Ong and Marshall, 1979; Ashmun et al., 1982; Schellner et al., 1982; Salzman, 1985; D’Hertefeldt and Jónsdóttir, 1994; Zhang et al., 2002; Liu et al., 2007). Clonal integration is referred to as the physiological integration that takes place among different ramets in clonal plants, and includes resource translocation and information communication (Dong, 1996a,b, 2011). In terms of its evolutionary implications and adaptive significance, it is one of the most specific and important characteristics possessed by clonal plant species (Dong, 2011). Owing to clonal integration, the patterns of materials translocation within clonal plants are more complicated than those of non-clonal plants (Dong, 1996a,b, 2011).

Spatiotemporal heterogeneity of environments is pervasive in all natural habitats (Wiens, 1976; Smith and Vrieze, 1979; Turkington and Klein, 1991; Turkington et al., 1991; Magyar et al., 2007). External resources like light, water, and mineral nutrients, which are essential for plants, and environmental conditions such as temperature and moisture, are distributed heterogeneously at various scales, including at scales relevant to individual plants (Dong, 2011). How to cope with environmental heterogeneity is one of the foremost problems that plants have to solve (Magyar et al., 2007). Connected ramets of clonal plants often experience different environmental conditions: for example, some ramets may be located in microsites with an abundant resource supply and/or without a particular stress or disturbance, while other ramets of the same genet are located in unfavorable microsites with scarce resources and/or severe stresses or disturbances. If there is resource translocation or information sharing within a clonal plant, donor ramets will help resource-poor or otherwise adversely situated ramets to alleviate their shortages and/or to resist stress and disturbances, resulting in an increase in the performance of the recipient ramets, and sometimes for that of the whole plant (Song et al., 2013). Most studies on clonal plants that spread through horizontal connecters (for example, stolons or rhizomes) support this notion. It is widely accepted that clonal integration – as one of the most important adaptive functional traits of clonal plants in dealing with environmental heterogeneity – came into being during the long-term evolution of plant clonality (Dong, 2011).

In this paper, we present a comprehensive review of research on clonal integration. We place a particular focus on plant environmental-adaptation strategies that derive specifically from clonal integration and also consider the ecological consequences of these strategies. We also propose perspectives for future research on this topic.

## Clonal Integration as a Core Concept for Clonal Plant Research

In an effort to obtain an overall picture of the importance of the topic of clonal integration in research relating to “clonal plants,” we downloaded the full metadata (including tiles, authorship, abstracts, keywords, keywords plus, and citations, as well as cited references) of clonal plant-related papers from the ISI Web of Science Core Collection for the publication period of 1900–2016. We used the search key terms “clonal plant^∗^,” and excluded any document defined as note, correction/addition, editorial material or meeting abstract. In total, 1369 articles (henceforth, the “clonal plants dataset”) were obtained.

Terms parsed from articles can be seen to be representative of key focal points of scientific research (Cui et al., 2012). We used CiteSpaceIII software^1^ to generate knowledge maps of key words from the clonal plants dataset. CiteSpace, developed by Chaomei Chen at Drexel University, USA (see Chen, 2004, 2006; Chen et al., 2010), is a Java application that supports visual exploration with knowledge discovery based on bibliographic information, and has been widely used in various research domains over the last decade. We set the relevant CiteSpace parameters as follows: time slicing (years 1991 and 2016), years per slice (3 years), term source (title, abstract, and author keywords, and keywords plus), and node type (keywords/term). The reason we chose 1991 as the beginning year for time slicing is that prior to that time only a few (less than three) relevant papers were published each year.

**FIGURE 1:**
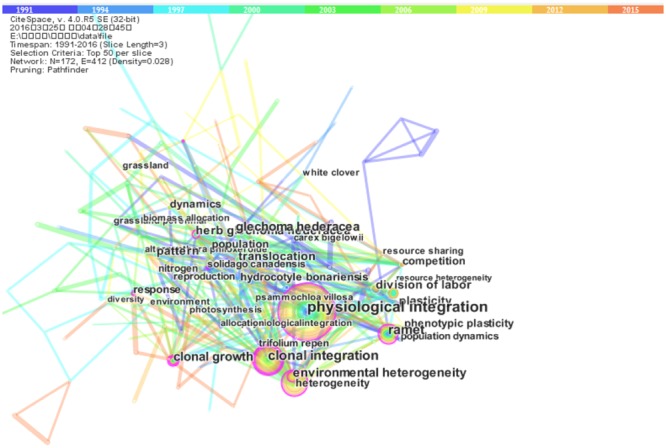
**The visualization of highly frequently occurring terms extracted from articles with the topic of “clonal plants” indexed by Thomson Routers.** This knowledge map was done using CiteSpace.

**Figure 1** provides an overview of these terms, from which we can see that the terms of “clonal integration” and “physiological integration” occupy the core position. Environmental heterogeneity is another highly frequent term (**Figure 1**), which is related very closely with clonal integration.

## Ecological Strategies to Deal with Environmental Heterogeneity

### Integrating Local and Non-local Responses

The traits of plants in nature are determined by both genetics and environmental conditions. The term plasticity is used to describe the fact that a given genotype may express a range of phenotypes under different environmental conditions (Schlichting, 1986; Bradshaw and Stettler, 1995; de Kroon et al., 2005). That is to say, plastic responses of plants are induced by variations in their surrounding environments, such as the quality and or quantity of essential resources (de Kroon et al., 2005). Such variations in environmental factors are normally present at multiple scales in many natural ecosystems (Hutchings and Wijesinghe, 1997). For example, the quality and/or quantity of soil resources in Southern Quebec forests vary remarkably over a distance of only 10s of centimeters (Lechowicz and Bell, 1991). In chalk grassland, light availability varies at a scale of 1 cm (Silvertown, 1981). This means that the physically interconnected ramets of a clonal plant may experience a variety of microhabitats with different environmental conditions (Stuefer and Hutchings, 1994; Hutchings and Wijesinghe, 1997; Hutchings et al., 1997; de Kroon et al., 2005). Ramets are the behavioral unit of plastic responses in clonal plant species (de Kroon et al., 2005). However, the local plastic responses of a ramet can be modified via clonal integration. In other words, responses can be induced both by the local environmental conditions that the ramets experience and by exchanges with interconnected ramets (Dong, 1996a,b; de Kroon et al., 2005). According to de Kroon et al. (2005), clonal integration changes the plant’s plastic responses at the ramet-level in three different ways, enhancing responses to local environment for a ramet, diminishing local responses to local environment for a ramet, and triggering new responses that are absent without integration. According to the model described by Magyar et al. (2007), such kind of plasticity is beneficial the most to plants that grow in spatially heterogeneous, yet temporally stable environments. When environmental conditions change at a short temporal scale, a non-plastic strategy becomes favorable (Magyar et al., 2007). However, most of previous studies on relationship between integration and plastic responses of clonal plants did not distinguish the particular effects exerted by spatial variation and temporal variation. Separating the effects of spatial and temporal environmental variation could provide more insights.

### Division of Labor

If the distribution patterns of two or more essential resources are not consistent within the area that a clonal plant covers, it is hard to determine whether or not the microhabitat of a ramet is good. Occasionally, the microhabitat is abundant in one resource but scarce in another, causing the availabilities of different resources to be negatively correlated with each other within patches (Alpert and Stuefer, 1997; Magyar et al., 2007). For instance, clonal herbs in the forest edge often experience negatively correlated light and soil resource conditions. For example, some ramets grow in a microsite with high light intensity and low soil nutrient availability, while interconnected ramets have complementary resource conditions, with low light intensity and high soil nutrient status (Cook, 1983; Alpert and Mooney, 1986; Friedman and Alpert, 1991; Stuefer and Hutchings, 1994; Stuefer et al., 1994, 1996; Alpert and Stuefer, 1997). This resource distribution pattern is called “reciprocal patchiness of resources (RPR)” (Alpert and Stuefer, 1997; Yu et al., 2002). Because it is least costly for a plant to acquire resources when they are abundant (Bloom et al., 1985; Stuefer et al., 1996), ramets of clonal plants subjected to an RPR-environment tend to specialize in acquiring whichever resource is locally abundant (Hutchings et al., 1997). Complementary resources are then reciprocally exchanged among the ramet systems in a bi-directional manner via clonal integration (Evans and Whitney, 1992; Alpert and Stuefer, 1997; van Kleunen and Stuefer, 1999). In doing so, each locally abundant resource can be acquired to the maximum extent by the whole genet This behavior, called “division of labor induced by environments,” is unique to clonal plants, and only exists among interconnected ramets (Stuefer, 1998). Non-clonal plants or single ramets usually display specialization for the uptake of the most limited resource (specialization for scarcity; Dong, 1996a,b; Hutchings and Wijesinghe, 1997).

Another phenomenon known as “developmentally programmed division of labor” (Stuefer, 1998) takes place in various rhizomatous plant species that can form large clonal fragments consisting of a number of interconnected ramets in environments with poor soils (Callaghan, 1976, 1984; Jónsdóttir and Callaghan, 1988, 1990; Schmid, 1990; Jonsson et al., 1996; Jónsdóttir and Watson, 1997). When clonal fragments grow, the above-ground parts (shoot) of some older mature ramets may die, but the below-ground parts (root system) can remain alive, while connected young ramets have active leaves yet have poorly developed roots. The leafless mature ramets often specialize in uptake of water and nutrients from the soil, while the young ramets are specialized for capturing light. At the same time, integration allows extensive translocation of light and light, water and nutrients through the entire fragment. Thus, either type of ramet undertakes specific responsibilities for acquiring different resources, and enjoys the benefits from resource sharing (Stuefer, 1998). This kind of “labor division among different-aged ramets” seems not to be associated with the spatial distribution pattern of resources, but is rather inherently driven by the plant’s own programmed development (Stuefer, 1998).

### Foraging for Resources

*Glechoma hederacea* was shown to have short internodes, copious branching, and a great number of ramets with large leaves when growing under either nutrient-rich soil (Slade and Hutchings, 1987c) or high light intensity conditions (Slade and Hutchings, 1987b); which could indicate that clonal plants forage for key resources (Salzman, 1985; Slade and Hutchings, 1987a,b,c; Alpert, 1991; Evans, 1991, 1992; Kelly, 1992; Hutchings and de Kroon, 1994; Bergamini et al., 2001). Some other herbs were observed to behave similarly, such as *Ranunculus repens* (Ginzo and Lovell, 1973), *Ipomoea phillomega* (Penalosa, 1983), *Trifolium repens* (Harper, 1983), and *Solidago canadensis* (Hartnett and Bazzaz, 1983). When subjected to heterogeneous environmental conditions, clonal plants prefer to inhabit favorable patches and avoid unfavorable patches via selective placement of ramets (Slade and Hutchings, 1987a,b,c; Kelly, 1992; Adam et al., 2003). The adaptive implication of this is quite clear: in order to ensure performance and fitness of the whole clone, more resource-acquiring structures are produced in resource-rich sites to exploit the abundant essential resources in an intensive way. However, foraging responses are more obvious when clones expanded from resource-poor microsites toward resource-rich microsites. For instance, when individual clones of *G. hederacea* in low-light and low-nutrient conditions grew into high-light and high-nutrient conditions, the morphology of the newly developed ramets changed significantly. In comparison, the change in ramet morphology was less conspicuous when clones grew from the rich into poor conditions (Slade and Hutchings, 1987a). Likely, foraging responses (e.g., development of more ramets with larger leaves and longer petioles) were reinforced when *G. hederacea* developed from low light patches toward high light ones; these responses were likely suppressed if expanded from high light patches toward low light ones (Wijesinghe and Hutchings, 1996). A recent meta-analysis conducted by Xie et al. (2014) showed that some typical foraging traits of clonal plants such as spacer length, specific-spacer length, branch intensity, and branch angle responded to light intensity but did not respond to nutrient or water availability. They also found that stoloniferous plants foraged resources more significantly than rhizomatous plants (Xie et al., 2014).

### Coordinating Inter-ramet Relations

Clonal integration contributes greatly to the establishment of new ramets, leading to a genet or a clonal fragment composed of a great number of interconnected ramets (Zahner and Debyle, 1965; Hartnett and Bazzaz, 1983; Bullock et al., 1994). Since each ramet is a potentially fully functional individual, a genet or a clonal fragment can be seen as a population of ramets (Herben et al., 1994). Ramet coexistence may have negative consequences in the form of inter-ramet for resources. Based on the fact that many clonal invertebrates avoid kin competition within taxonic families (Sebens, 1984; Ayre and Grosberg, 1995; Ishii and Saito, 1995) and the fact that plants tend to place their roots away from their neighbors (Brisson and Reynolds, 1994; Schenk, 1999), Holzapfel and Alpert (2003) evaluated the hypothesis that physiological integration could enable clonal plants to minimize interference among connected ramets of the same genet via root segregation. Their experiment with *Fragaria chiloensis* showed that in connected ramet pairs, less root biomass was placed between the pair, and more was placed on the sides away from each other, in comparison to separated ramet pairs. Due to root separation, the performance of the genets increased considerably (Holzapfel and Alpert, 2003). It is likely that physiological integration between connected ramets resulted in the avoidance of self-competition in *F. chiloensis* (Holzapfel and Alpert, 2003). This experiment provided clear evidence to support the hypothesis that integration in at least some clonal plant species can facilitate cooperation and reduce inter-competition among ramets thereby enhancing the overall performance of the genet or of the clonal fragment. *Carpobrotus edulis* was also observed to avoid competition for resources via physiological integration by adjusting the biomass allocation to roots among connected ramets (Roiloa et al., 2014). However, whether this phenomenon is a broadly general among most species or is specific to a given species will require further studies.

## Ecological Consequences of Clonal Integration

### Alleviating Resource Shortage

Interconnected ramets of a genet in clonal plant species are often located in different microhabitats, resulting in a source-sink gradient along ramets in terms of resource quality (Dong et al., 2007). The resources acquired by ramets growing in favorable microhabitats can be transported to ramets growing where resources are scarce, thanks to clonal integration. Clonal integration can therefore alleviate local shortages of resources for ramets in poor microsites (Hutchings and Wijesinghe, 1997). In this case, recipient ramets benefit directly from the import of resources that they lack, while those same resources are relatively ample for donor ramets. On the other hand, the donor ramets may have to bear loss to some extent due to the export of their resources. Using cost-benefit analysis, many empirical studies have found that the benefits frequently outweigh the costs.

The biomass of ramets, the number of newly born ramets and the number of seeds of *Hydrocotyle bonariensis* in low N availability, are all significantly increased when connected to ramets with a high N supply. Even when clonal growth and reproduction of N-rich ramets were impaired, the overall benefit was still higher than the cost (Evans, 1988). Disconnected clones of *Potentilla simplex* weighed significantly less than connected clones under under heterogeneous nutrient conditions, but differed little under homogeneous conditions (Wijesinghe and Handel, 1994). In addition, a field-based experiment on a rhizomatous grass, *Psammochloa villosa*, supported the hypothesis that water translocation could alleviate water shortage often experienced by *P. villosa* (Dong and Alaten, 1999). For *Potentilla reptans* and *Potentilla anserina* in heterogeneous light quality conditions, specifically when there was a connection between ramets in full-light and ramets in shaded patches, shade did not negatively impact the performance of either of the ramets (Stuefer et al., 1994). Clonal integration has been shown to alleviate different types of resource shortage, such as shading, nutrient depletion and drought in many clonal plant species in numerous experiments (Guttridge, 1959; Ginzo and Lovell, 1973; Hartnett and Bazzaz, 1983; Salzman, 1985; Alpert, 1991, 1999a,b; de Kroon et al., 1996; Dong and Alaten, 1999; Alpert et al., 2003). By combining a large body of empirical evidence, Magyar et al. (2007) found that a modular cooperation strategy was most advantageous when environmental conditions varied spatially. Since the resources necessary for plant growth (such as light, water, and mineral nutrients) are distributed unevenly in all habitats, clonal integration is viewed as being beneficial for clonal plant species.

### Buffering Stress and Disturbance

In addition to the uneven distribution of essential resources, clonal plant species may also suffer various local biotic and abiotic stresses and disturbances. Since some case studies can fall under both the topics of “resource shortage” and “stressful environment,” e.g., water shortage or drought stress, we limited “stress” in the present review to those adverse conditions that were not directly caused by the lack of essential resources. ‘Disturbance’ here refers to a change in environmental conditions that disrupt the ecosystem, community, or population structure and bring about a change in resources, substrate availability, or the physical environment (Pickett and White, 1985). Salzman and Parker (1985) found that ramets under salt stress connected with unstressed ramets accumulated more biomass than ramets connected with other stressed ramets in *Ambrosia psilostachya*. In the sandy grasslands of Inner Mongolia, China, a series of experiments to test the hypothesis that clonal integration may help local native plant species to withstand frequently occurring stresses and disturbances, such as sand burial, wind erosion, and grazing (Yu et al., 2001, 2004, 2009; Liu F.H. et al., 2006; Liu et al., 2009; Xu et al., 2012). Their results showed that the connection through rhizomes conferred a considerable increase in performance to *P. villosa* during both sand burial and wind erosion. This positive effect was more obvious when heavy sand burial and sand erosion were imposed on the plant (Yu et al., 2004, 2008). Similarly, another dominant rhizomatous semi-shrub, *Hedysarum laeve*, and a stoloneferous herb, *P. anserina*, both benefited from clonal integration when subjected to sand burial (Yu et al., 2001; Liu F.H. et al., 2006). In addition, when *Bromus ircutensis* and *P. villosa* were subjected to heavy clipping (simulating grazing), clonal integration was found to function as an additional compensatory mechanism, greatly improving the performance of both species (Liu et al., 2009). When considering the simultaneous occurrence of both trampling and defoliation caused by grazing, clonal integration did not impact the response to defoliation, but did alleviate the trampling-induced damage to the root-suckering clonal tree *Populus simonii* (Xu et al., 2012). From the above results, it is reasonable to conclude that clonal integration indeed plays a key role in the long-term persistence of clonal plant species in the inland dune regions of northern China. In a few cases, though, this has not been consistently found. For example, in *Fargesia qinlingensis*, a bamboo species known for being a primary food of panda bears, clonal integration was shown to not be a compensatory response to herbivore feeding (Wang et al., 2007). Similarly, rhizome severing (cutting off clonal integration) did not significantly affect rhizome growth, ramet growth, or vegetative bud outgrowth of the ramet population in *Leymus chinensis* (Wang et al., 2004). One explanation could be that the experimental duration was not long enough to observe the real functions of clonal integration. Regardless, most of the existing case studies empirically suggest that clonal integration is an adaptive strategy, conferring advantages to clonal plant species to improve stress and disturbance tolerance.

### Increasing Competitive Ability

Given that clonal integration can increase the performance of clonal plants in a range of habitats, it seems to be advantageous for successful competition over non-clonal plants (van Groenendael et al., 1996; de Kroon and van Groenendael, 1997; Yu et al., 2010). However, many previous studies did not find that clonal integration strongly contributes to the interspecific competition ability of clonal plant species. Schmid and Bazzaz (1987) reported that cutting off the connection of *Aster* had very little influence on their intraspecific competition capacity, neither did for *Solidago*. Similar results were observed in *Brachypodium pinnatum* and *Carex flacca* (de Kroon et al., 1992). Based on the finding that the clonal tree *Populus tremuloides* did not profit from a rhizome connection when invading the native prairie, Peltzer (2002) suggested that clonal integration might be functional for exploiting patchy resources or tolerating stressful environments rather than for improving the competitive ability of clonal plant species (Salzman, 1985; Cain, 1994; Evans and Cain, 1995; Shumway, 1995; Brewer and Bertness, 1996; Dong, 1996a,b, 1999; Stuefer et al., 1996; Stoll and Schmid, 1998; Dong and Alaten, 1999; Yu et al., 2002, 2004, 2009; Zuidema et al., 2007). Wang et al. (2011) found that clonal integration enhanced the disturbance and drought resistance ability rather than the competitive ability of the rhizomatic *Eremosparton songoricum*. Pennings and Callaway (2000) compared the role of clonal integration in six salt marsh plant species and found that clonal integration was most important for plants invading stressful habitats, moderately important for plants invading sites with neighbor plants clipped, and least important for plants invading habitats with intact vegetation. These results were strongly in support of Peltzer’s (2002) conclusions. An invasive plant *Alternanthera philoxeroides* was also shown to benefit little from a stolon connection in invading a population of *Schedonorus phoenix* (Wang et al., 2008). In contrast, a few studies found that clonal integration promoted the competitive abilities in some clonal plants. The response of *S. canadensis* ramets to interspecific competition was affected by the ramet connection: connected ramets of *S. canadensis* responded more or less equally in their shoot growth, reproduction, and clonal growth while separated ramets exhibited large differences when grown together with different neighboring species (Hartnett and Bazzaz, 1985). The notion of benefits from clonal integration also holds true for an aquatic plant *Vallisneria spiralis*, for which stolon connection improved its ability to invade vegetated habitats (Xiao et al., 2011).

According to previous empirical evidence, we can say that the positive effects of clonal integration on the competitive ability of clonal plants are not as common as were once expected, but are very likely species-specific and strongly rely on environmental conditions. We propose that, when a clonal plant encounters competitors, it rapidly grows and forages to occupy new adjacent microhabitats, rather than competing directly. A meta-analysis compiling all existing case studies may provide more insights.

### Maintaining Community Biodiversity and Productivity

Whether and how the effects of physiological integration on clonal plant species are scaled to the community level is another interesting question (Oborny et al., 2000, 2012; Wilsey, 2002; Yu et al., 2009, 2010; Eilts et al., 2011). However, even though a tremendous amount of research has focused on the individual level, much less has attempted to measure the consequences of clonal integration on ecological processes at the community level (Eilts et al., 2011). Among the limited community-level studies, two coherent aspects were examined. One is whether the effects of clonal integration on genet growth can be translated to influence the productivity of the community (Wilsey, 2002; Yu et al., 2010). This is particularly relevant in the case of communities where clonal plant species are dominant and produce most biomass (Wilsey, 2002; Yu et al., 2009, 2010; Eilts et al., 2011). In the Serengeti grassland that has a high frequency of perennial rhizomatous and stoloniferous plants, all connecters between ramets severing significantly reduced the net primary productivity at a community level, suggesting that plants grew better when ramets remained connected (Wilsey, 2002). The other question, arguably more important, is whether the effects at the individual clonal plant species level further impact the species composition and diversity of a community. Spatial heterogeneity in soil resources can facilitate a species’ coexistence by enhancing the diversity of a community (MacArthur, 1972). Clonal plants, especially those that spread extensively, are thought to have the capacity to override fine-grain spatial heterogeneity (Hutchings and de Kroon, 1994) and thereby even the heterogeneity out via extensive resource translocation within the ramet network (Gough et al., 2002). Therefore, it has been supposed that clonal integration can impair the positive effect of resource heterogeneity on plant species richness. In a field experiment carried out over 6 years, rhizomatous clonal plants were found to have a strong negative effect on species richness (Eilts et al., 2011). In particular, the effect was strengthened when the spatial scale of nutrient heterogeneity within the scale at which rhizomatous clones could potentially integrate across resource patches (Eilts et al., 2011). This may result both from physiological integration and from the high competitive ability of clonal plants. Further study will be needed to disentangle the particular effects of clonal integration and those competitive ability traits *per se*. Experiments that keep the rhizome connected or not can be used to explore the particular effects resulting from clonal integration. Of note, in the Mu Us Sandland of northern China, rhizome connection neither increased growth of the dominant half-shrub *Hedysarum laeve* nor exerted any conspicuous influence on species composition (Yu et al., 2010).

The provisionary conclusions drawn from a limited number of cases cannot be generalized. More studies dealing with effects of clonal integration on community processes, particularly productivity, species coexistence, and diversity, will be needed.

### Enhancing Invasiveness and Invasibility

Plant invasion has become a significant threat to biodiversity for environments and economies, both globally and locally (Mack et al., 2000; Liu et al., 2005; Pysek and Richardson, 2010). There are a considerable number of invasive plant species that are capable of vigorous clonal propagation, and their invasiveness may be related to clonal integration (Liu J. et al., 2006; Yu et al., 2009). Many studies have shown that clonal integration can enhance plant invasion success in alien plants (Reichard and Hamilton, 1997; Liu et al., 2008; Aguilera et al., 2010; Roiloa et al., 2010, 2014). Song et al. (2013) compiled 84 case studies covering 57 taxa, to provide a synthetic analysis of the effects of clonal integration on the performance of clonal plants. The results showed that clonal taxa for which recipient ramets in unfavorable patches benefited more from integration, were also more invasive on a global scale. A few more recent experiments provided additional support. You et al. (2014a) found that the invasive clonal plant *A. philoxeroides* benefits from clonal integration more than the co-occurring native species *Jussiaea repens*, suggesting that the invasiveness of *A. philoxeroides* may be closely related to clonal integration in heterogeneous environments. They also found that clonal integration can help *A. philoxeroides* to respond to defoliation (You et al., 2014b). The non-native invasive taxa *Typha angustifolia* and *T. x glauca* benefited more from increased maternal resource availability than the native congeneric counterpart *T. latifolia*, which strongly suggested that clonal integration confers advantages for the invasion of *T. angustifolia* (Elgersma et al., 2015). Physiological integration of resources might improve the establishment of juvenile ramets of *Ludwigia hexapetala* across variable light environments during early colonization and thus contribute to its invasiveness (Gover et al., 2015). Similar results were also obtained by Wolfer and Straile (2012), Tuya et al. (2013), Roiloa et al. (2014), and Liu et al. (2016), but see Peltzer (2002).

Despite the existing evidence that clonal integration contributes to the invasive ability of clonal plants, what if any role it plays when clonal plants invade new habitats remains unclear. It is possible that the relationship(s) between clonal integration and invasion success is species-specific and/or stage-dependent. Considering that clonal integration does not necessarily improve the competition ability of clonal plants (see Increasing Competitive Ability), clonal integration might support the survival and growth of new ramets of invasive clonal plants at their introduction stage (but see Liu et al., 2010).

## Conclusion and Future Research

Since pioneer studies that began in the 1970s (Qureshi and Spanner, 1973), clonal integration has attracted considerable attention in the field of plant ecology. There is a large body of literature exploring the mechanisms of physiological integration between ramets in clonal plant species, and its ecological and evolutionary consequences have been widely considered. The previous studies arrived at the common viewpoint that clonal integration allows translocation of materials within a whole or partial clone. It can help clonal plants adjust the plastic responses, helps clonal plants avoid possible inter-ramet competition, and favor stressed or damaged ramets. Overall, clonal integration can enhance the performance of clonal plant species at the ramet, or sometimes at the genet level(s), especially in the context of environmental variation (Dong, 2011). Recent studies have provided new insights on this topic, suggesting that research on clonal integration is far from over. Here, we propose four possible research directions for open discussions:

(1) Du et al. (2009) found that arbuscular mycorrhizal fungi (AMF) reduced the effects of physiological integration in *Trifolium repens*. It provides the first evidence for interactions between colonization by AMF and effects of physiological integration in a clonal plant (Du et al., 2009). Since symbiotic associations between AMF and plant roots are common in natural environments (Gosling et al., 2006), it is necessary to take such interactions into consideration in future research. Results are expected to provide more insights on ecological importance of clonal growth in the spatial and temporal composition of plant communities.
(2) Dong et al. (2015) found that clonal integration increased performance in a homogeneous resource-rich environment when connected ramets of *A. philoxeroides* differed in external resource uptake ability. The sibling ramets always developed in a successive order along the runner (stolon or rhizome), and resource uptake ability therefore usually differs from one ramet to another. Thus, the result produced from this study is likely not just an isolated case. We need more evidence to test this idea.
(3) Theoreticaly, clonal plants have to incur large costs, including potentially evolving slower owing to reduced sexual reproduction, risking the accumulation of mutations because absence in recombination and the possibility to create genetic variation in offspring, and the lack of the benefits of DNA repair mechanisms in comparison of plants with sexual reproduction (Douhovnikoff and Dodd, 2015). However, clonal plants are widespread throughout the plant kingdom and are found in diverse habitats (Price and Marshall, 1999), and many clonal plants both bear clonal growth and sexual reproduciton. To explain the contradiction between the theory and the facts, Douhovnikoff and Dodd (2015) proposed that epigenetic mechanisms might help clonal plants outweigh the evolutionary costs, and clonal plants may use epigenetic acclimation over long stretches of evolutionary time to adapt to environmental variation. What role, if any, that clonal integration may playin epigenetics processes is also one of the far-reaching and promising topics for future research.
(4) New findings by Ye et al. (2016) suggest that the resources from a donor microsite (here referring to the microsite where donor ramets are) could be translocated within a clonal network and then released into recipient microsites (here referring to the microsite where recipient ramets are) and these resources could subsequently be used by neighbor plants, resulting in resource redistribution at a community level. The findings of this study raise the very novel question of whether clonal integration could facilitate water and nutrient cycling, and therefore have implications for the whole ecosystem. More empirical evidence is needed to address this fascinating question.

## Author Contributions

FL, JL, and MD: proposed the outline of the manuscript. FL: wrote and revised the manuscript; MD: made a final check of the manuscript.

## Conflict of Interest Statement

The authors declare that the research was conducted in the absence of any commercial or financial relationships that could be construed as a potential conflict of interest.

## Footnotes

1 http://cluster.cis.drexel.edu/~cchen/citespace/
